# Anti-Angiogenic Therapy: Albumin-Binding Proteins Could Mediate Mechanisms Underlying the Accumulation of Small Molecule Receptor Tyrosine Kinase Inhibitors in Normal Tissues with Potential Harmful Effects on Health

**DOI:** 10.3390/diseases9020028

**Published:** 2021-04-10

**Authors:** Nicolae Ghinea

**Affiliations:** Research Center, Translational Research Department, Curie Institute, Tumor Angiogenesis Team, 75005 Paris, France; nicolae.ghinea@curie.fr or nicolae.ghinea@inserm.fr

**Keywords:** albumin-binding proteins, albumin-drug complexes, angiogenesis, anti-angiogenic therapy, endocytosis, transendothelial transport, endothelial FSHR

## Abstract

**Simple Summary:**

In the last five decades the tumor microvascular endothelium has been, and still remains, an object of sustained interest in biomedical research. This interest is undoubtedly connected with the importance of blood microvessels in cancer cell proliferation, tumor growth, and metastasis. Depriving a tumor of its oxygen and nutrients by preventing the formation of new vessels with anti-angiogenic drugs including small molecule receptor tyrosine kinase inhibitors (RTKIs) is a common treatment in oncology. However, resistance to treatment, insufficient efficacy, and high toxicity limit the success of this antivascular therapy. Cellular and molecular mechanisms mediated by several albumin-binding proteins (ABPs) expressed in normal tissues and organs seem to be responsible for the side effects and toxicity associated with this type of anti-angiogenic therapy.

**Abstract:**

Anti-angiogenics currently used in cancer therapy target angiogenesis by two major mechanisms: (i) neutralizing angiogenic factors or their receptors by using macromolecule anti-angiogenic drugs (e.g., therapeutic antibodies), and (ii) blocking intracellularly the activity of receptor tyrosine kinases with small molecule (M_r_ < 1 kDa) inhibitors. Anti-angiogenics halt the growth and spread of cancer, and significantly prolong the disease-free survival of the patients. However, resistance to treatment, insufficient efficacy, and toxicity limit the success of this antivascular therapy. Published evidence suggests that four albumin-binding proteins (ABPs) (gp18, gp30, gp60/albondin, and secreted protein acidic and cysteine-rich (SPARC)) could be responsible for the accumulation of small molecule receptor tyrosine kinase inhibitors (RTKIs) in normal organs and tissues and therefore responsible for the side effects and toxicity associated with this type of cancer therapy. Drawing attention to these studies, this review discusses the possible negative role of albumin as a drug carrier and the rationale for a new strategy for cancer therapy based on follicle-stimulating hormone receptor (FSHR) expressed on the luminal endothelial cell surface of peritumoral blood vessels associated with the major human cancers. This review should be relevant to the audience and the field of cancer therapeutics and angiogenesis/microvascular modulation-based interventions.

## 1. Introduction

In animal models for human cancer, angiogenesis is a prerequisite for tumor growth beyond 2 mm^3^ [1]. The endothelial cell (EC) proliferation is stimulated by various tumor secreted angiogenic factors including vascular endothelial growth factor (VEGF) [2], platelet-derived growth factor (PDGF) [3], fibroblast growth factor (FGF) [4,5], and angiopoietins [6]. Angiogenic factors act via paracrine signaling when they are released by tumor and stromal cells or when they are mobilized from the extracellular matrix (ECM) [5]. The information conveyed by the angiogenic factors is transmitted to transmembrane tyrosine kinase receptors that are expressed on the abluminal surface of ECs lining the pre-existent blood vessel neighborhood of a tumor implant. The activation of these ECs causes degradation of the endothelial basal membrane and of the ECM, which facilitates the EC migration and proliferation, and a tube formation resulting in new vascular sprouts [7]. As the sprouting of new blood vessels from the pre-existing ones is a sine qua non condition of tumor progression and metastasis, inhibiting this process by using anti-angiogenesis therapeutic agents may halt the growth and spread of cancer [8,9].

Anti-angiogenic therapy is based on the concept that tumor vessels can be selectively targeted without affecting the normal organs’ vasculature [10], which is characterized by an extensive coverage with pericytes that can control the quiescent endothelial phenotype [11]. The anti-angiogenic drugs currently used in cancer therapy target the proliferating tumor ECs by two major mechanisms: neutralizing angiogenic factors or their receptors by using macromolecule anti-angiogenic drugs (e.g., therapeutic antibodies) or blocking the receptor tyrosine kinases intracellularly with small molecule (M_r_ < 1 kDa) receptor tyrosine kinase inhibitors (RTKIs) bound to albumin [12]. While some anti-angiogenic drugs inhibit the pathways that affect the initiation of tumor angiogenesis (e.g., the VEGF pathway), others impair the maintenance of the angiogenic process (e.g., the FGF pathway).

The encouraging study data on angiogenic factor-targeted therapies and their mechanisms of action in preclinical models have led to the translation of these therapies to the clinic (e.g., VEGF-targeted therapies) [13,14,15]. However, anti-angiogenic therapies have shown limited efficacy in the clinical management of various types of cancer. One reason for this seems to be the difference between the highly proliferative experimental tumors supported by a new immature highly angiogenic microvasculature that grows rapidly (from 2 to 7 days) [16], and human tumors (e.g., prostate cancer) that grow over years and are mainly supplied with oxygen and nutriments by pre-existing more mature (i.e., less angiogenic and less permeable) blood vessels [17], co-opted by cancer cells.

In animal models the sprouting angiogenesis is the main mechanism by which tumors acquire a rich microvasculature [1]. The experimental tumors may consist of 40% ECs [18] and the majority of ECs in the neighborhood of a tumor implant are proliferating cells, responding well to the antiangiogenic treatments. By contrast, in many human tumors the microvasculature generally represents only a small fraction of the tumor mass [19] and a minority of ECs are proliferating: 0.15% for prostate cancer [20], 0.7% for clear cell renal cell carcinoma [21], 2.2% for breast cancer [22], 9.9% for colorectal cancer [23], and 10.6% for hepatocellular carcinoma [24]. Moreover, in many cases human tumors can induce their own microvasculature not only by sprouting angiogenesis but also through non-angiogenic mechanisms involving non-proliferating cells (e.g., co-option of pre-existing normal vessels, recruitment of bone-marrow-derived stem cells, and vasculogenic mimicry) that explain the resistance of tumors to conventional anti-angiogenic treatments [25,26,27,28,29,30,31]. These non-angiogenic mechanisms and the resistance mechanisms to common molecularly targeted agents (e.g., angiogenic redundancy, angiogenic dormancy, tumor metabolic adaptation) could explain the frequent inefficacy of the anti-angiogenic treatments with small molecule RTKIs and monoclonal therapeutic antibodies (reviewed in [32,33]).

## 2. How Anti-Angiogenics Reach Their Target in Cancer: Cellular and Molecular Mechanisms Involved in the Accumulation of Drugs in Tumors

Because the receptors for angiogenic factors are expressed on the abluminal (tissue-facing) plasma membrane of ECs, the small molecule RTKIs and the anti-angiogenic antibodies must cross the endothelial layer of tumor blood vessels. Although small enough to diffuse easily inside tumor tissues (calculated spherical diameter < 1 nm) (https://nanocomposix.eu/pages/molecular-weight-to-size-calculator; accessed on 9 April 2020), the small molecule RTKIs (Table 1) should suffer from a relatively short circulating half-life that limits their therapeutic potential. However, being highly hydrophobic molecules, the RTKIs bind to plasma proteins, especially to albumin (the most abundant plasma protein, which exhibits an average half-life of 19 days), improving their pharmacokinetic profile. Albumin is a protein consisting of 585 amino acids and has a molecular mass of ~ 66 kDa. Because one third of amino acids have an electrical charge (83 positive (lysine and arginine) and 98 negative (glutamic acid and aspartic acid)) albumin has a negative net electric charge (isoelectric point, pI = 5.2) and a high solubility in aqueous solutions at physiological pH [34]. In human plasma, the average plasma concentration is 42 g/L. The presence of 17 covalent disulphide bonds makes human albumin very stable to changes in pH, heat exposure, and denaturing solvents. Based on its inherent biochemical and biophysical properties, the albumin is considered an ideal platform for cancer therapeutic administration [34,35,36].

**Figure 1:**
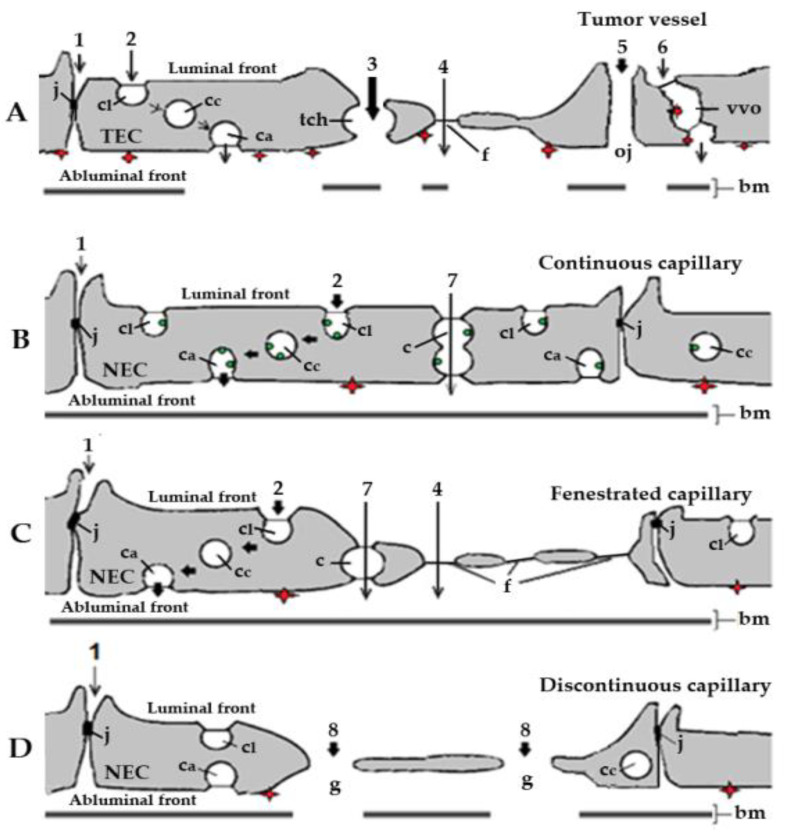
Schematic representation of the basic types of blood capillaries in cancer and healthy organs and tissues. Organelles and routes potentially involved in the transendothelial transport of small molecule receptor tyrosine kinase inhibitors (RTKIs) bound to plasma albumin. (**A**). Tumor capillary. Effective and efficient transport of RTKI-albumin conjugates by diffusion via large transendothelial channels (tch, pathway 3) and open junctions (pathway 5) towards interstitial space of cancer tissues. By contrast, the actual participation of tight junctions (pathway 1), caveolae (pathway 2), fenestrae (pathway 4), and vesiculo-vacuolar organelles (pathway 6) in the transport of anti-angiogenic agents from plasma to the subendothelial space in tumors should be very limited. (**B**). Continuous capillary. Caveolar transport (pathway 2)—possibly the main transport mechanism of RTKIs bound to albumin mediated by albumin-binding proteins (ABPs) in continuous capillary endothelia of normal organs and tissues (e.g., skeletal and cardiac muscles, in fat tissue, connective tissue, arterial capillaries of the lung, and in the skin). (**C**). Fenestrated capillary. Caveolar fluid phase transport (pathway 2; possibly the main transport mechanism of RTKI-albumin conjugates and anti-angiogenic antibodies in fenestrated capillary endothelia of normal organs and tissues). The transport through the diaphragmed fenestrae (pathway 4) should make a minor contribution to the transport of anti-angiogenics due to their negative charges (e.g., fenestrated capillaries in healthy kidney, digestive tract mucosa, and all endocrine glands). (**D**). Discontinuous capillary. Diffusion through the endothelial gaps (pathway 8; effective and efficient transport but only in liver, spleen, and bone marrow), highly dependent on the concentration of both RTKIs and anti-angiogenic antibodies in blood. The red diamonds represent the angiogenic receptor tyrosine kinases (RTKs) exposed on the abluminal (tissue) front of capillary endothelia. In tumor capillaries, the angiogenic RTKs (the targets of anti-angiogenic agents) are overexpressed. The green dots represent albumin-binding sites (ABPs) associated with caveolae in healthy continuous capillaries. bm, basal membrane; c, channel; c_a_, abluminal caveolae; c_c_, cytoplasmic caveolae; c_l_, luminal caveolae; f, fenestra; g, gap; j, junction; NEC, normal endothelial cell; tch, large transendothelial channel; oj, open junction; TEC, tumor endothelial cell. Adapted with permission from ref. [41]. Copyright 1995 Journal of Endocrinology.

Electron microscopy analysis of blood vessels associated with tumor xenograft mouse models for human cancer [37,38,39] has identified caveolae, fenestrae, and vesiculo-vacuolar organelles, three subcellular structures that should be involved in the transendothelial transport of anti-angiogenic molecules (Figure 1A). However, due to the presence of widened (up to 2 µm in diameter) intercellular junctions between tumor endothelial cells [37], and particularly of many large transendothelial channels [40], the actual participation of caveolae, fenestrae, and vesiculo-vacuolar organelles in the transport of anti-angiogenic agents from plasma to the subendothelial space in tumors is very limited. Diffusion mechanisms should therefore allow an extensive and effective transport of small molecule RTKI-albumin conjugates and anti-angiogenic therapeutic antibodies from blood to cancer tissues. The higher the dose of RTKIs and antibodies, the better their efficacy.

An enhanced permeability of tumor blood vessels, the overexpression of secreted protein acidic and cysteine-rich (SPARC), a matrix-associated protein that binds albumin, and a lack of lymphatic drainage should result in high accumulation of therapeutic antibodies and albumin-RTKI complexes in solid tumors but not into normal tissues and organs [33]. However, factors such as focal necrosis, high interstitial pressure, low microvascular pressure, and the presence of a tumor microvasculature induced by non-angiogenic mechanisms delay extravasation of these anti-angiogenics [42]. This is why an undesirable enhanced transendothelial transport of albumin-RTKI complexes could occur at the level of normal microvasculature, especially in patients with tumors that acquired their microvasculature through non-angiogenic mechanisms. Therefore, the accumulation of albumin-RTKI complexes in healthy organs that may express ABPs could cause serious adverse side effects and toxicity with time [43,44,45].

## 3. Diffusion and ABP-Mediated Mechanisms Could Underlie the Accumulation of RTKIs Bound to Albumin in Healthy Organs and Tissues

### 3.1. Endothelial Cell Pathways that May Be Involved in Transport of RTKIs Bound to Albumin

In healthy organs and tissues the exchanges of molecules between the blood and tissues take place at the level of capillaries and postcapillary venules. The main constituent of the wall of capillaries and venules is the endothelium, a single layer of thin flattened ECs that lines their lumens. Morphological studies with the use of tracers visible at the electron microscopic level (reviewed in [46]) have shown that the microvascular ECs are polarized and linked to each other by junctional complexes (tight, adherens, gap) which ensure the continuity and mark the transition between the luminal (blood) and abluminal (tissue) fronts. In contrast to the tumor endothelia, the microvascular endothelium in healthy organs and tissues constitutes a selective barrier for the bidirectional exchanges of macromolecules between the plasma and the interstitial fluid [47,48,49]. Four structures involved in the transendothelial transport of macromolecules in healthy organs and tissues have been defined: intercellular junctions [50,51], caveolae [52], transendothelial channels [53], and endothelial fenestrae [54]. Concerning the presence or absence of fenestrae and discontinuities, three types of capillary endothelia have been classified as continuous (nonfenestrated) (Figure 1B), fenestrated (Figure 1C), and discontinuous (sinusoids) (Figure 1D) (review in [35]). Continuous capillaries are the most common type, and are found in the brain, skeletal and cardiac muscles, in fat tissue, connective tissue, arterial capillaries of the lung, and in the skin.

The endothelial junctions in capillaries of healthy organs and tissues have a low degree of permeability for macromolecules (>2 nm in diameter) [55,56], especially. By contrast, 30% of postcapillary venules and muscular venules exhibit loosely organized endothelial junctions and appear to be permeable to macromolecules having a diameter lower than 5.5 nm [55]. During inflammation, the permeability of the venular junctions is markedly increased and this increased permeability is accompanied by the appearance of numerous gaps (up to 1 µm) between the cells [57] through which plasma molecules including anti-angiogenics drugs (e.g., small molecule RTKIs and therapeutic antibodies) may reach by diffusion healthy tissues and organs.

All ECs contain a population of caveolae (small plasma membrane invaginations 50–70 nm in outer diameter) that move freely from the luminal to the abluminal front of the endothelial cells carrying plasma molecules. The caveolae can take up a bulk of plasma (fluid phase transport) or can carry molecules that have been adsorbed onto the caveolar membrane either electrostatically [58,59,60] or via cognate-specific binding sites [61,62,63,64,65]. The latter mechanism could be involved in the transendothelial export of small molecule RTKIs bound to albumin from blood towards normal tissues in peripheral capillaries with a continuous endothelium (see below).

Transendothelial channels (produced by fusion of several caveolae [66] and/or vesicular invaginations [67]) are open on both the apical and abluminal fronts of the capillary endothelium of normal organs and tissues (Figure 1B,C). They behave as sieves with openings of 20–40 nm. However, the very rare occurrence of such transendothelial channels [66] suggests that their actual participation in the transport of small molecule RTKIs bound to albumin from plasma towards the interstitial space of normal organs and tissues is very limited.

The endothelial fenestrae (Figure 1C), round openings (up to 70 nm in diameter), are found in the fenestrated capillaries of endocrine glands, kidneys, choroid plexus, ciliary bodies, and the mucosa of the gastrointestinal tract [68]. The high density of proteoglycans of high negative charge associated with the luminal aspect of their diaphragms [69,70] should limit the passage through the fenestrae of anionic anti-angiogenics like the small molecule RTKIs bound to albumin.

In the discontinuous capillary endothelia of liver, spleen, and bone marrow, ECs contain large gaps (>100 nm in diameter; Figure 1D) and, in consequence, the endothelium does not impede the transport of anti-angiogenics. Therefore, as in the case of tumor microvessels (see above), diffusion mechanisms allow an extensive and effective accumulation of anti-angiogenics in liver and hematopoietic tissues.

### 3.2. ABP-Mediated Accumulation of RTKIs Bound to Albumin in Healthy Organs and Tissues Could Be Responsible for Side Effects and Toxicity

**Figure 2:**
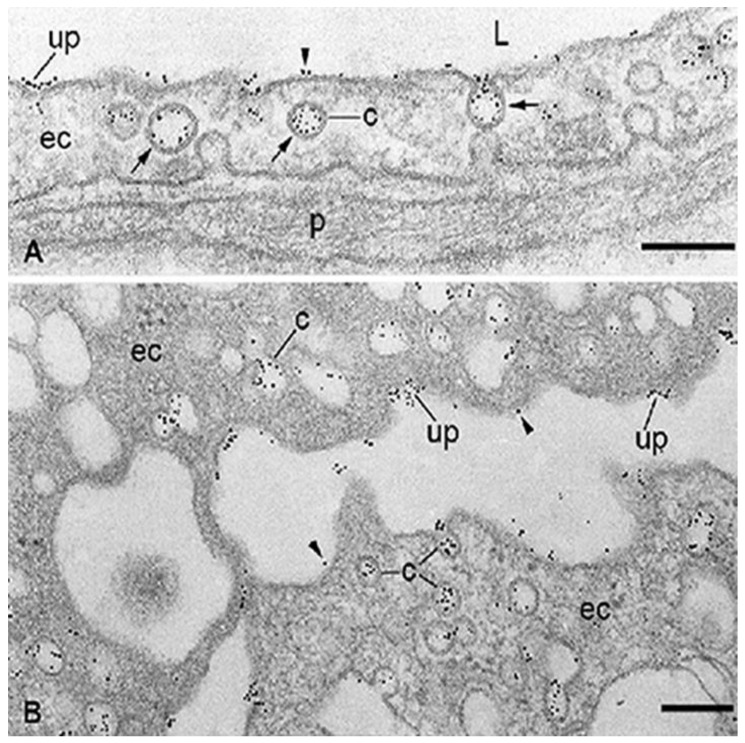
Localization at the electron microscope level of specific binding-sites for albumin associated with the capillary endothelium in healthy adipose tissue (**A**) and microvascular endothelial cells (ECs) freshly isolated from rat epididymal fat (**B**). The binding of bovine serum albumin (BSA)-Au5_nm_ conjugate is restricted to the membrane of caveolae (c), predominantly in an adsorptive pattern (arrows) and some uncoated pits (up). Some particles of BSA-Au5_nm_ conjugate rarely appear on plasma membrane proper (arrowhead). ec, endothelial cell; L, capillary lumen; p, pericyte. The scale bar represents 200 nm in both panels. Adapted with permission from ref. [62]. Copyright 1988 Journal of Cell Biology.

The albumin-RTKI complexes form rapidly in the blood and travel through the circulation until they enter the blood vessels of the tumor. Ideally, these complexes should neither interact with, nor be transported through, the microvascular endothelia of healthy organs and tissues. Unfortunately, this is not the case because the drugs that are tightly associated, conjugated, or fused with albumin are kept in circulation and delivered to organs and tissues at a rate and in a similar manner to that of plasma albumin. The capillary endothelium of normal lung, heart, diaphragm, skeletal muscle, and adipose tissue contains three specific albumin-binding glycoproteins (gp18, gp30) [62,63,64], and gp60/albondin [64] restricted mainly to caveolae (Figure 2). Therefore, the accumulation of these drugs bound to albumin could occur not only in tumors but also in normal tissues and organs which, like the tumors, contain SPARC [71]. SPARC is an ABP secreted by various cells whose main function is to mediate interactions between cells and their extracellular matrix during morphogenesis, tissue remodeling, and angiogenesis [72]. Approximately 80% of the total extravascular pool of albumin is equally divided between muscles and the skin [35]. While gp60 is involved in the transendothelial transport of native albumin [64], gp18 and gp30 also act as scavenger receptors that mediate the high affinity endocytosis and degradation of conformationally and chemically modified albumins [73]. Gp18 and gp30 are distributed ubiquitously, being found in the lung, liver, kidney, fat, heart, muscle, brain, adrenal glands, and pancreas [74]. Cultured cells expressing gp30 and gp18 such as the endothelial cells, cardiomyocytes, smooth muscle cells, and fibroblasts are able to bind, internalize, and degrade albumin conformationally modified by endogenous and exogenous ligands including the tyrosine kinase inhibitors. Importantly, chemically modified albumin has a 1000-fold higher affinity for both gp18 and gp30 than native albumin [74], and therefore native albumin should not inhibit the binding, transendothelial transport, accumulation, and degradative process of RTKI-albumin complexes in healthy organs and tissues.

Studies on healthy rats have shown that [^68^Ga]ABY-028, an albumin-binding domain protein-based imaging tracer for positron emission tomography, binds in vivo to albumin, acquires albumin circulatory behavior, and accumulates in liver, spleen, and in muscle tissues [75]. Similar data were published by Park and collaborators [76] who noticed effective accumulation of ^64^[Cu]-labeled-click chemistry-based albumin nanoplatform conjugates in normal lung, liver, kidney, intestine, and heart with an over four-fold higher uptake than the tumor at 1 h post-injection. Zhong and collaborators [77] showed effective accumulation of anlotinib (an oral RTKI targeting VEGFR, PDGFR, FGFR, and c-Kit; Table 1) in female tumor-bearing mice. The level of tumor exposure to the compound increased as the dose increased. However, after an oral dose of 3 mg/kg of anlotinib the level of tumor exposure was approximately 13.5, 8, and 6.5 times smaller than the level of liver, lung, and kidney, respectively. These data indicate that only the RTKI-albumin complexes that escape the accumulation in normal organs and tissues have the opportunity to interact with the tumors. Because all tissues catabolize albumin [78], the degradation of RTKI-albumin complexes internalized via the caveolar ABPs (i.e., gp18 and gp30) in the cellular lysosomes of healthy organs and tissues may result in a sudden, undesired increase in the free fraction of RTKIs that may accumulate in the cytoplasm of cells and cause adverse effects and toxicity [79,80,81,82,83,84], especially in cases of long-term use of these drugs. The competitive displacement between high-dose administered RTKIs and endogenous compounds such as fatty acids, amino acids, metal ions, hormones, bile acids, and toxic metabolites that share the same binding sites on albumin could also have harmful effects on the body. Moreover, high doses of RTKIs may increase tumor aggressiveness and metastasis [85,86,87]. The accumulation of small molecule RTKIs in normal organs should not be specific for antiangiogenic drugs. Similar ABP-mediated mechanisms are likely to be involved in the accumulation of other therapeutic agents (e.g., chemotherapeutics) bound to albumin in all the healthy tissues and organs where ABPs are expressed. The adverse effects of anti-angiogenesis therapy with small molecule RTKIs include hypertension, diarrhea, fatigue, proteinuria, hand and foot syndrome, thrombocytopenia, and skin and hair discoloration. The combination of small molecule RTKIs bound to albumin with chemotherapeutic agents was thought to improve efficiency and reduce adverse effects on patients. However, this treatment failed to show a survival benefit since the toxicity of the small molecule RTKIs was additive to that of cytotoxic agents [88,89].

In conclusion, the published data suggest that, although albumin improves the pharmacokinetic profile of small molecule inhibitors, it is the binding of drug-albumin complexes to ABPs expressed in healthy tissues and organs of the patients that could be responsible for the side effects and toxicity of RTKIs with time. The development of more specific carriers for small molecule inhibitors than albumin is necessary to enhance efficacy and to reduce the occurrence of adverse effects and toxicities.

## 4. Perspectives

Anti-angiogenic therapy is effective if it can slow down, stop, or reduce the size of tumors at the microscopic level. However, the efficacy of this type of cancer therapy is substantially compromised by the inability of anti-angiogenic drugs to kill tumor cells, especially those located at the periphery of the tumor mass [90,91]. At this level tumor cells obtain oxygen and nutrients from unaffected peritumoral blood vessels that make connections between the normal circulatory system and tumor core vessels. Therefore, disruption of peripheral and peritumoral vessels may provide a new strategy in cancer therapy. Very high affinity anti-receptor monoclonal antibodies could succeed in cancer therapy [92]. Several antibodies raised against angiogenic targets are actually used in anti-angiogenic therapy (reviewed in [93]). Injected intravenously, these antibodies must cross the vascular endothelium, bind to the respective antigens (angiogenic factors or receptors for the angiogenic factors) and block their signaling from being effective. However, as mentioned above, not all tumors recruit their vasculature by sprouting angiogenesis.

The monoclonal antibodies raised against endothelial cell receptors exposed on the luminal surface of tumor blood vessels, but absent at the level of blood vessels in healthy tissues, should offer an opportunity for biomarker-specific delivery of therapeutic agents. Studies from our laboratory [94,95,96,97,98] and others [99,100,101] have indicated that this is the case for the follicle-stimulating hormone receptor (FSHR), a G-protein-coupled receptor. The FSH/FSHR signaling is known to generate activated Gq/11 protein that induces VEGFR-2 signaling in ECs, even in the absence of VEGF [102]. This effect may be responsible for the peripheral angiogenesis noticed in human cancer [21].

**Figure 3:**
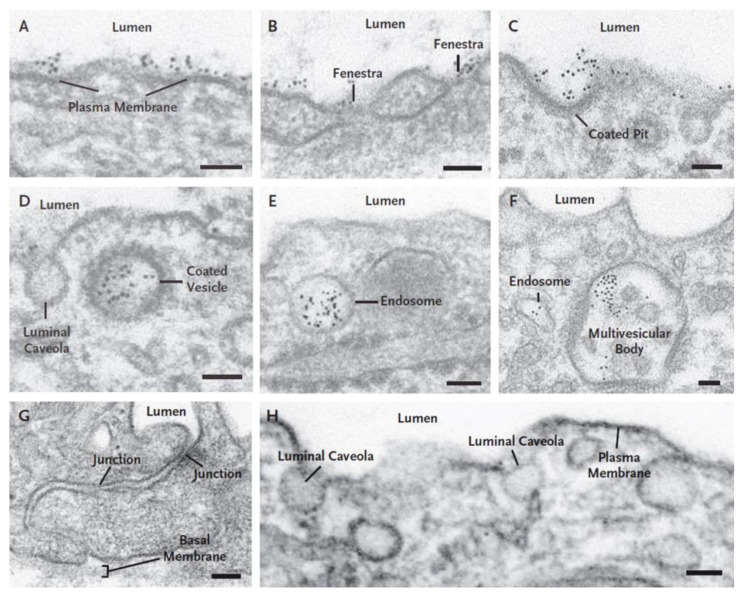
Receptor-mediated mechanism underlying the internalization of mouse monoclonal anti-FSHR antibody coupled to colloidal gold in ECs of tumor blood vessels associated with tumors generated by the prostate cancer line LNCaP in nude mice. After 20 min of perfusion of FSHR-Au5_nm_ the tracer was bound to the plasma membrane (**A**), to diaphragms of fenestrae (**B**), to luminal clathrin-coated pits (**C**), and internalized in clathrin-coated pits (**D**), early endosomes (**E**), and multivesicular bodies (**F**). No tracer was internalized in the caveolae open to the lumen of the tumor blood vessel (**C**,**D**). No tracer particles crossed the endothelial barrier through the junctions (**G**). No tracer particles were seen associated with the microvascular ECs in the normal lung (**H**). Scale bars represent 50 nm in panels (**A**–**F**) and H, and 100 nm in panel G. Reprinted with permission from ref. [94]. Copyright 2010 Massachusetts Medical Society.

The generation of a highly specific antibody (FSHR323) directed against the extracellular domain of the receptor [103] has allowed the study of the expression of FSHR in various pathophysiological conditions in humans and animal models. FSHR is expressed in 11 human solid tumor types [93], 11 types of soft-tissue sarcomas [98], and in the majority of metastatic tumors [95]. FSHR (as mRNA and protein) is present in ECs of solid tumors, most frequently at the periphery of the tumors [94]. By using immunoelectron microscopy we demonstrated that, in a mouse tumor model for human prostate cancer, FSHR is present on the luminal surface of the tumor ECs and can specifically bind and internalize anti-FSHR antibodies coupled to colloidal gold particles delivered in the circulation [94] (Figure 3).

To target the FSHR-positive peritumoral vessels a new approach can be developed: peritumoral vascular infarction. The proposed strategy represents an alternative to the current anti-angiogenic approaches, which, when targeting angiogenic receptors expressed on the abluminal endothelial surface of tumor blood vessels, have not been successful in securing long-term remission of disease. An innovative strategy to kill tumors could be the use of either a bispecific antibody able to engage T cells (this bispecific antibody will recognize FSHR and CD3) or FSHR-specific chimeric antigen receptor-T cells. In the latter case, as a binding moiety for the immunoreceptor, either highly specific monoclonal anti-human FSHR antibodies [104] or FSH (the natural ligand of FSHR) can be used [105,106]. Indeed, these cells should easily recognize FSHR expressed on the luminal surface of peripheral and peritumoral endothelia in direct contact with blood. Since FSHR is a common marker of tumor vessels, this strategy should in principle be applicable to a wide range of solid tumor types and of soft tissue sarcomas. In healthy organs and tissues, the expression of FSHR at the level of ECs is restricted to testis and ovaries but at a much lower concentration than that observed in tumors. Sertoli and granulosa cells are the only cells in the organism expressing a high concentration of FSHR. However, these cells are not directly accessible to the blood stream. Moreover, some damage to these cells may be acceptable for advanced cancer cases in patients over the reproductive age.

## Figures and Tables

**Table 1:** Small molecule anti-angiogenics used in cancer therapy ^a,b,c^.

RTKI ^c^	Molecular Weight	Albumin Binding	RTK
	(Da)	(%)	
Anlotinib	407.4	93.0	VEGFR, PDGFR, FGFR
Apatinib	397.5	92.4	VEGFR, PDGFR
Axitinib	386.5	99.0	VEGFR, PDGFR
Cabozatinib	501.5	99.7	VEGFR
Cediranib	450.5	99.8	VEGFR, PDGFR
Leflunomide	270.2	99.8	PDGFR
Lenvatinib	523.0	99.0	VEGFR
Nilotinib	529.5	93.9	PDGFR
Pazopanib	437.5	99.9	VEGFR, PDGFR
Ponatinib	532.6	99.0	VEGFR, FGFR
Regrorafinib	482.0	99.0	VEGFR
Sorafenib	464.8	99.5	VEGFR, PDGFR
Sunitinib	398.5	95.0	VEGFR
Tivozanib	454.9	99.0	VEGFR
Vandetanib	475.4	90.0	VEGFR, EGFR

^a^ PubChem data (https://pubchem.ncbi.nlm.nih.gov/compound; accessed on 9 April 2020). ^b^ Upon administration these RTKIs target multiple RTKs including VEGFR, PDGFR, FGFR, and EGFR and may both inhibit angiogenesis and halt tumor cell proliferation. ^c^ Please note list is not exhaustive. VEGFR indicates vascular endothelial growth factor receptor; PDGFR, platelet-derived growth factor receptor; FGFR, fibroblast growth factor receptor, EGFR, epithelial growth factor receptor.

## Data Availability

Not applicable.
